# Comparative studies of salinomycin-loaded nanoparticles prepared by nanoprecipitation and single emulsion method

**DOI:** 10.1186/1556-276X-9-351

**Published:** 2014-07-15

**Authors:** Qin Wang, Puyuan Wu, Wei Ren, Kai Xin, Yang Yang, Chen Xie, Chenchen Yang, Qin Liu, Lixia Yu, Xiqun Jiang, Baorui Liu, Rutain Li, Lifeng Wang

**Affiliations:** 1 Nanjing Drum Tower Hospital, Clinical College of Nanjing Medical University, 321 Zhongshan Road, Nanjing 210008, China; 2 The Comprehensive Cancer Center of Drum Tower Hospital, Medical School of Nanjing University and Clinical Cancer Institute of Nanjing University, Zhongshan Road 321, Nanjing 210008, People's Republic of China; 3 Laboratory of Mesoscopic Chemistry and Department of Polymer Science and Engineering, College of Chemistry and Chemical Engineering, Nanjing University, 22 Hankou Road, Nanjing 210093, People's Republic of China

**Keywords:** Salinomycin, Nanoprecipitation method, Single emulsion method, Gelatinase, Drug delivery, Nanoparticles

## Abstract

To establish a satisfactory delivery system for the delivery of salinomycin (Sal), a novel, selective cancer stem cell inhibitor with prominent toxicity, gelatinase-responsive core-shell nanoparticles (NPs), were prepared by nanoprecipitation method (NR-NPs) and single emulsion method (SE-NPs). The gelatinase-responsive copolymer was prepared by carboxylation and double amination method. We studied the stability of NPs prepared by nanoprecipitation method with different proportions of F68 in aqueous phase to determine the best proportion used in our study. Then, the NPs were prepared by nanoprecipitation method with the best proportion of F68 and single emulsion method, and their physiochemical traits including morphology, particle size, zeta potential, drug loading content, stability, and in vitro release profiles were studied. The SE-NPs showed significant differences in particle size, drug loading content, stability, and in vitro release profiles compared to NR-NPs. The SE-NPs presented higher drug entrapment efficiency and superior stability than the NR-NPs. The drug release rate of SE-NPs was more sustainable than that of the NR-NPs, and in vivo experiment indicated that NPs could prominently reduce the toxicity of Sal. Our study demonstrates that the SE-NPs could be a satisfactory method for the preparation of gelatinase-responsive NPs for intelligent delivery of Sal.

## Background

Cancer stem cells (CSCs) represent a small proportion of cancer cells that exist in the cancer cell population, which drives tumor growth and recurrence [1,2]. And CSCs are found to be resistant to conventional cancer treatments, including chemotherapy and radiotherapy [3-6], which suggests that many cancer therapies, though killing bulks of tumor cells, may ultimately fail because they do not eliminate CSCs, which survive to regenerate new tumors. Alternatively, tumor drug-resistant cells induced by traditional chemotherapeutics also present cancer stem-like characteristics [7]. Therefore, novel therapeutic strategies specifically targeting CSCs are urgently needed.

Salinomycin (Sal) was originally used to kill bacteria, fungi, and parasites [8]. In 2009, Gupta et al. identified Sal by a high-throughput screening that could potentially be used to target breast CSCs, and it killed breast CSCs at least 100 times more effectively than paclitaxel in mice [9]. Since then, large amounts of studies have been conducted and indicated that Sal can effectively kill various types of cancer stem-like cells, for instance, colorectal cancer, pancreatic cancer, prostate cancer, and so on [10-12]. Thus, it is considered to be a potential anticancer drug for cancer therapy. However, for several reasons, Sal has never been established as a drug for human diseases until now. For example, owing to its poor aqueous solubility, it could not be administered by intraperitoneal injection without the aid of ethanol [13]. In particular, several reports and studies published in the last three decades revealed a considerable toxicity of Sal in different kinds of mammals including humans after accidental oral or inhalative intake [14-17]. To solve these problems, significant efforts have been done, and the widely used are nanoparticles (NPs) due to their satisfying traits.

Amphiphilic copolymer NPs consisting of hydrophilic and hydrophobic segments have drawn intensive attention in antitumor drug delivery systems since the 1990s [18-20]. They present unique characteristics as drug carriers including high drug encapsulation efficiency due to optimized drug solubility in the core [21], preferential accumulation in tumor tissue via enhanced permeation and retention (EPR) effect [22,23], the ability to prolong drug release [24] because of the protection of the drug against chemical and enzymatic degradation [25], and the capacity to reduce the systemic adverse effects and the risks of toxicity [26,27] with the purpose of minimizing the number of administrations for a better patient compliance [28]. Progress in this field has made it possible for NPs being able to deliver the drugs to the targeted tumor sites [29-31], which can increase the drug concentration in the tumor region and reduce the drug content in the normal region. This characteristic can reduce the side effects of the drugs to a large extent. In the present work, the intelligent gelatinase-stimuli NPs which are modified by inserting the optimal gelatinase-cleavable peptide (PVGLIG) between polyethylene glycol (mPEG) and polycaprolactone (PCL) segment (mPEG-Pep-PCL) [32] were set up to deliver Sal. Owing to their special structures, the NPs prepared from mPEG-Pep-PCL have their own characteristics, such as prolonged circulating time and accumulation in the tumor site by the EPR effect [33-35]. Since the gelatinases are known to be abundantly present in most tumors, once the NPs accumulate in the tumors, the mPEG-PCL conjugates will be cleaved at the certain site of the peptide which results in the dePEGylated NPs being retained in the tumor regions, effectively interacting with more tumor cells, increasing cellular uptake of NPs into cancer cells, and improving intracellular anticancer drug concentration.

By encapsulating Sal into the PEG-Pep-PCL NPs that are based on biodegradable polymers, its side effects can be reduced because of the EPR effects of copolymeric NPs, sustained release pattern, and gelatinase-triggered drug targeting. NPs can be formulated by using different preparation methods [36]. However, few studies have been done to evaluate the satisfying preparation method for Sal delivery by NPs. Thus, the aims of this work are to emphasize the relationship among the particle elaboration process, the nature of the particles, their size stability, and the encapsulated drug release and to choose the optimized way for Sal delivery. In our study, we confirmed the optimum preparation method for Sal-loaded NP preparation.

## Methods

### Synthesis of PEG-peptide conjugates

Two-hundred milligrams of methoxy-polyethylene glycol-NHS (mPEG-NHS, Jiankai Technology Co., Ltd., Beijing, China) and 26 mg of PVGLIG (HD Biosciences Co., Shanghai, China) were dissolved in 3 mL of dimethyl formamide (DMF) containing 3% triethylamine (Et3N) and stirred for 3 h at room temperature to prepare PEG-peptide conjugates. The unconjugated peptides were removed by dialysis in a 3,500-Da MWCO membrane (Greenbird, Shanghai, China) against deionized water for 24 h.

### Synthesis of PCL-COOH copolymers

The dichloromethane (DCM)-dissolved PCL (Mn13800, determined by proton nuclear magnetic resonance (^1^H NMR)) polymers were added with succinic anhydride, 4-dimethylamiopryidine (DMAP), and pyridine and then stirred at room temperature for 48 h. The resultant mixture was precipitated in ethyl ether and washed three times with methanol and then dried under vacuum to prepare the PCL-COOH.

### Synthesis of PCL-NH_2_ copolymers

The DMF-dissolved PCL-COOH polymers were added with DMAP, 1-ethyl-3-[3-dimethylaminopropyl] carbodimide hydrochloride (EDC), ethylenediamine and *N*-hydroxysulfosuccinimide sodium salt (NHS), and stirred at 37°C for 18 h. The mixture was precipitated in ethanol and dried under vacuum to prepare the PCL-NH_2_.

### Synthesis of PEG-Pep-PCL and PEG-PCL copolymers

The mixture of PCL-NH_2_ (0.002 mol), PEG-peptide conjugates (0.002 mol), DMAP (0.003 mol), EDC (0.002 mol), and NHS (0.002 mol) in DMF was stirred for 24 h at 32°C. The crude PEG-peptide-PCL copolymers were purified by dialysis (MWCO 13 kDa) for 24 h, lyophilized to powder, and stored at 4°C for further use.

### Nanoprecipitation method with different proportions of poloxamer 188 (F68) in deionized water (aqueous phase)

Different concentrations of surfactants were designed to select the optimal formulation, which could have long-term stability. Briefly, 5 mg of PEG-Pep-PCL copolymers was dissolved in 200 μL acetone, and then, the mixed solution was slowly added to five aqueous solutions (5 mL each) containing 0.125%, 0.25%, 0.5%, and 1.0% (*w*/*w*) F68 with quick stirring. The NPs formed and the solution turned to blue immediately. The remaining acetone was removed by rotary vacuum evaporation and the resulting solution was filtered to remove nonincorporated drugs.

### Sal NPs and blank NPs prepared by nanoprecipitation method with the best proportion of F68

After the optimal surfactant concentration had been determined, 5 mg of copolymer and 0.5 mg of Sal (China Institute of Veterinary Drug Control, Beijing, China) were dissolved in 200 μL acetone, and the mixture was added into 5 mL deionized water that contains the best proportion of F68 with quick stirring. The NPs formed and the solution turned to blue immediately. The remaining acetone was removed by rotary vacuum evaporation and the resulting solution was filtered to remove nonincorporated drugs. The blank NPs were produced in the same manner without adding Sal.

### Single emulsion method

The NPs loaded with Sal were prepared by a modified single emulsion method. Briefly, 20 mg of each copolymer and 2 mg Sal were dissolved in 1 mL of DCM. The mixture was emulsified in 3 mL of aqueous polyvinyl alcohol (PVA) solution at 3% (*w*/*v*) by sonication (XL2000, Misonix, Farmingdale, NY, USA) for 60 s (4 W) to obtain an o/w emulsion. This emulsion was then emulsified in 5 mL of aqueous solution containing 0.5% (*w*/*v*) PVA by sonication (XL2000, Misonix, USA) for 10 s (2.5 W). The w/o/w emulsion formed was gently stirred at room temperature in a fume hood until the evaporation of the organic solvent was complete. The resulting solution was filtered to remove nonincorporated drugs. Blank NPs were produced in the same manner without adding Sal.

### Size and zeta potential analysis of the NPs

Mean diameter and size distribution were measured by photon correlation spectroscopy (DLS) with a Brookhaven BI-9000AT instrument (Brookhaven Instruments Corporation, Holtsville, NY, USA). Zeta potential was measured by the laser Doppler anemometry (Zeta Plus, Zeta Potential Analyzer, Brookhaven Instruments Corporation, USA). All measurements were performed at 25°C. The values were calculated from the measurements performed at least in triplicate.

### Morphology studies

Morphological examination of the NPs was conducted using a JEM-100S transmission electron microscope (TEM, JEOL Ltd., Akishima-shi, Japan). One drop of NP suspension was placed on a copper grid covered with nitrocellulose membrane and air-dried before observation.

### Drug loading content and encapsulation efficiency

To determine the Sal-loaded NPs’ drug loading content, 1 mL of the NPs was dried in the oven. Then, Sal was redissolved in 3 mL 95% ethanol and centrifuged. The supernatant derivatized with vanillin (Accelerating Scientific and Industrial Development thereby Serving Humanity, Shanghai, China) in an acidic medium at 72°C for 40 min. The derivatization mixture was determined by the ultraviolet absorption at the wavelength of 527 nm, a strong absorption band of Sal with reference to a calibration curve on a Shimadzu UV3100 spectrophotometer (Shimadzu, Hadano, Japan).

Drug loading content and encapsulation efficiency were obtained by the following equations:

$$\begin{array}{l} \mathrm{Drug}\;\mathrm{loading}\;\mathrm{content}\;\left(\%\right)\\ \;=\frac{\mathrm{Weight}\;\mathrm{of}\;\mathrm{the}\;\mathrm{drug}\;\mathrm{in}\;\mathrm{nanoparticles}}{\mathrm{Weight}\;\mathrm{of}\;\mathrm{the}\;\mathrm{nanoparticles}}\times 100\%\; \end{array}$$

$$\begin{array}{l} \mathrm{Encapsulation}\;\mathrm{efficiency}\;\left(\%\right)\\ \;=\frac{\mathrm{Weight}\;\mathrm{of}\;\mathrm{the}\;\mathrm{drug}\;\mathrm{in}\;\mathrm{nanoparticles}}{\mathrm{Weight}\;\mathrm{of}\;\mathrm{the}\;\mathrm{feeding}\;\mathrm{drug}}\times 100\%\; \end{array}$$

### Stability evaluation

Sal-loaded NPs and blank NPs were kept at room temperature. Particle sizes were determined by DLS every 2 days for 15 days to evaluate stability.

### In vitro release of Sal-loaded NPs

In vitro release of Sal from NPs was investigated by a dialysis method. Briefly, a volume of 1 mL NP solution was sealed in the dialysis bag (molecular weight cutoff 14,000 Da). The dialysis bag was immersed into a 5-mL 0.01 M pH 7.4 PBS solution containing 0.5% Tween 80 at 37°C in a constant temperature shaker. At predetermined time points, aliquots were withdrawn from the beaker and replaced with equal volume of the media. The Sal content in the release medium was determined by pre-HPLC (derivatized with 2,4-dinitrophenol (DNP, Accelerating Scientific and Industrial Development thereby Serving Humanity, Shanghai, China) in an acidic medium at 55°C for 30 min in a C18 column (Agilent Technologies, Ltd., Santa Clara, CA, USA), with the mobile phase consisting of methanol:1.5% aqueous acetic acid = 93:7, the flow rate of 1 mL/min, the column temperature of 25°C, the injection volume of 20 μL, and the detector at a wavelength of 392 nm). All the experiments met the sink condition and were repeated in triplicate.

### In vivo safety studies of Sal-loaded SE-NPs

All animal studies were performed in compliance with guidelines set by the Animal Care Committee at Drum Tower Hospital, Nanjing, China. 8BC nudes (18 to 22 g, 4 to 5 weeks, male, Animal Care Committee at Drum Tower Hospital, Nanjing, China) were embedded with 1 × 1-cm tumor pieces subcutaneously on the right axilla. Tumor dimensions were measured with vernier calipers, and the volumes were calculated as follows: Tumor volume (mm^3^) = Width^2^ × Length / 2. When the tumors reached 100 mm^3^ (designated as day 1), the animals were randomized into the following treatment groups: free Sal (4 mg/kg), free Sal (8 mg/kg), and Sal NPs prepared by the single emulsion method (SE-NPs) (8 mg/mL). The number of surviving mice was counted to preliminarily evaluate the safety of Sal SE-NPs in vivo.

### Statistical analysis

Statistical analyses of data were done using Student's *t* test. The data are listed as mean ± SD, and values of *P* < 0.05 were accepted as a statistically significant difference.

## Results and discussion

### Copolymer synthesis and characterization

**Figure 1:**
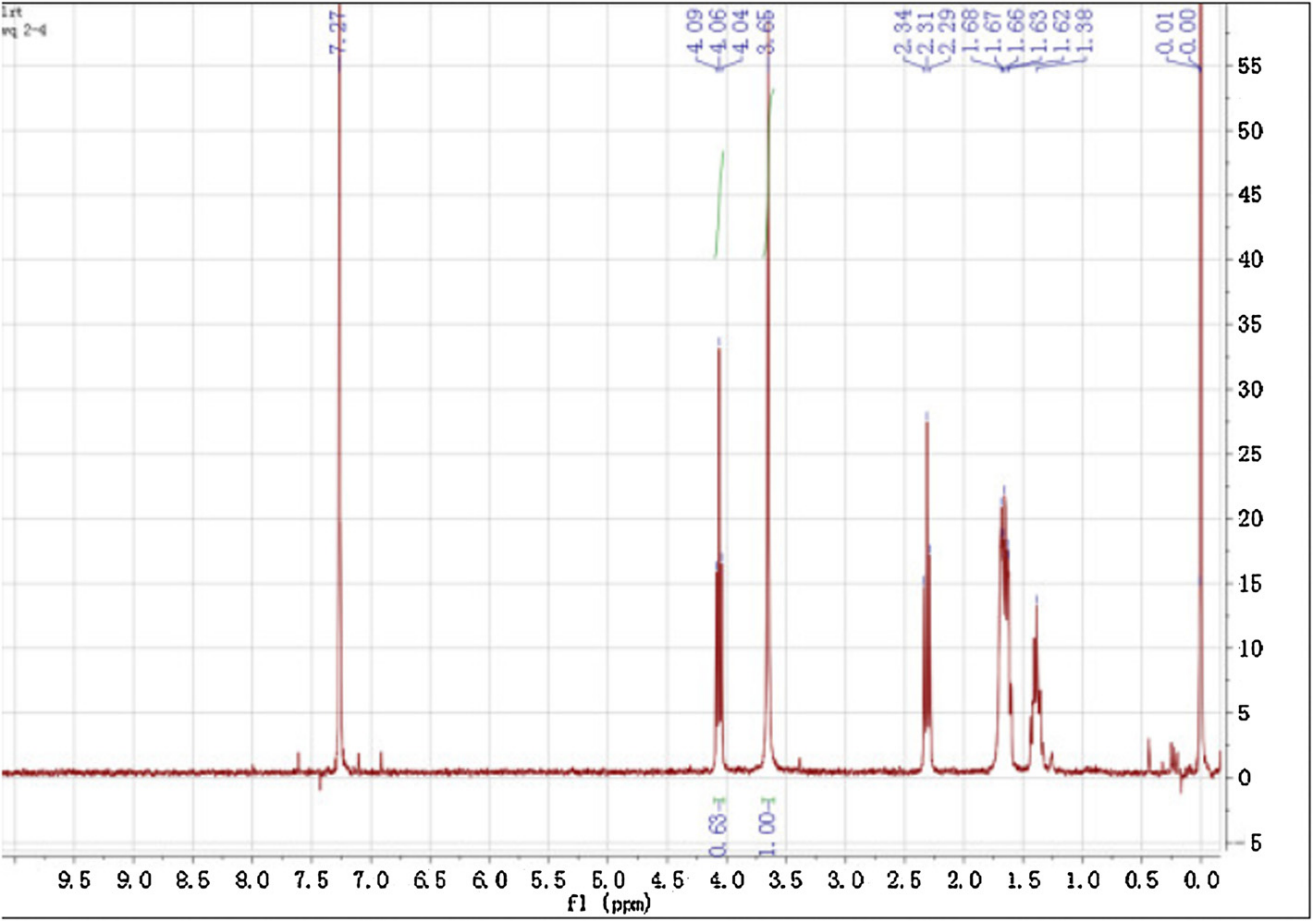
^**1**^**H nuclear magnetic resonance spectra (300 MHz, 25 μC) of PEG-Pep-PCL in CDCl**_**3**_**.**

The mPEG-Pep-PCL copolymers were synthesized as described in the ‘Methods’ section. Figure 1 shows the ^1^H NMR spectra of mPEG-Pep-PCL copolymers in trichloromethane (CDCl_3_), indicating that the peptide was successfully conjugated into the copolymer. The mole ratio of hydrophilic block to hydrophobic block (mPEG/PCL) in mPEG-Pep-PCL copolymer was about 0.63 based on the integral ratio of -CH_2_-O- (4.044 ppm) in PCL segment to -CH_2_-CH_2_-O (3.5 ppm) in mPEG segment from ^1^H NMR measurement. Thus, the number-average molecular weight (Mn) of the resulting mPEG-Pep-PCL copolymer was determined to be approximately 24,038 (Figure 1).

### Determination study of the surfactant concentration

**Figure 2:**
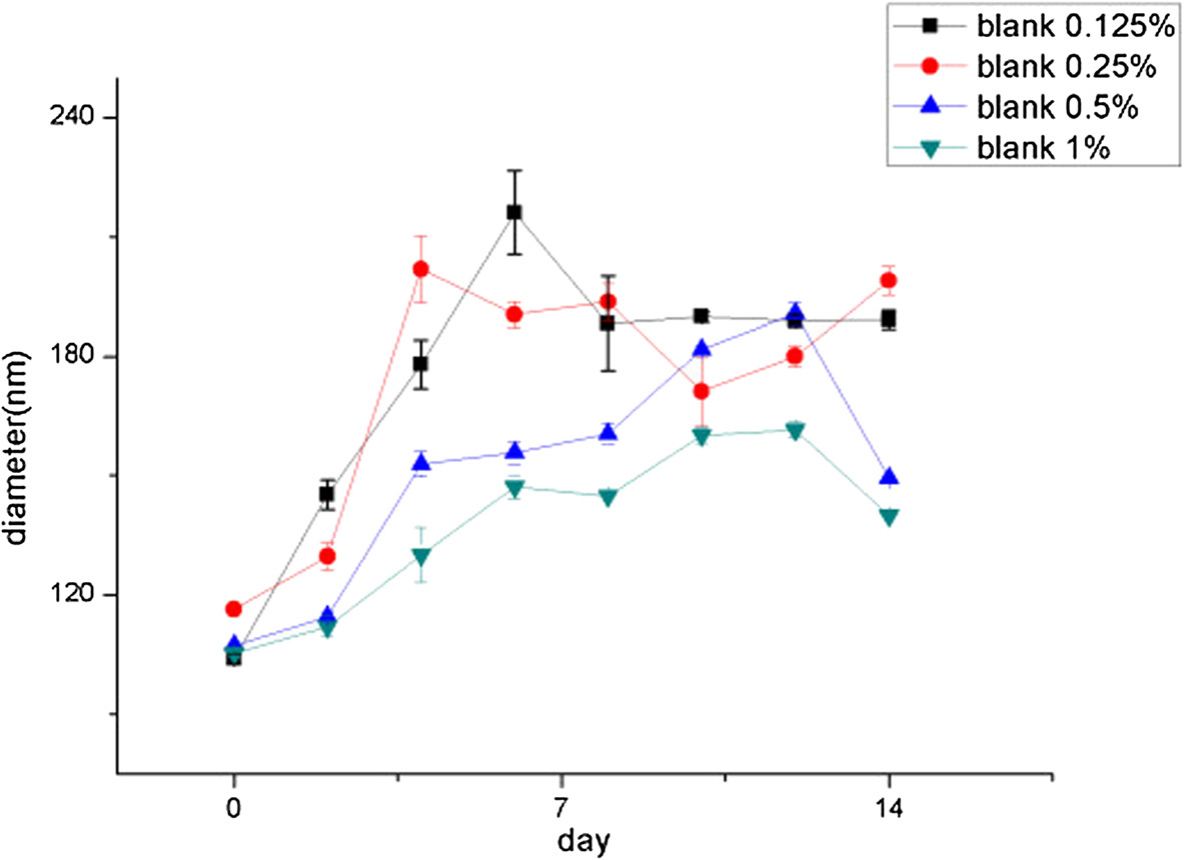
The stability of NPs with different concentrations of F68.

In the nanoprecipitation method, the surfactant is not really involved in the formation of NPs; it acts as a stabilizer keeping the particles' stability. It was found that a fourfold increase of the size was observed for the particles without surfactant. Instead, only small variations in size were observed in 2 months for those prepared with the surfactant [37]. Nevertheless, there were no studies about the relationship between the concentration of F68 and NP stability. In our study, four proportions were tried and the results are listed in Figure 2. According to the results, the stability of NPs increased in accordance with the increase of F68 concentration, and when the F68 proportion was 1%, the optimal NP formulation was obtained with the smallest particle size (mean particle size about 137.56 nm) and a more stabilized property. Based on this screening, 1% turned out to be the best proportion for NP suspensions.

### Size and morphology studies of NPs prepared by the two methods

**Figure 3:**
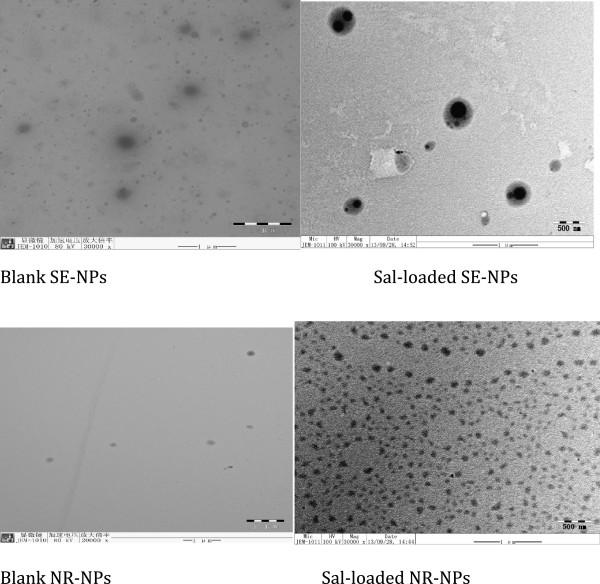
The TEM images of Sal NPs prepared by different methods.

The particle size and size distribution of these four NPs in aqueous solution were determined by DLS, and the results are displayed in Table 1. As we can see, the particle sizes of the NPs prepared by the nanoprecipitation method (NR-NPs) and SE-NPs were approximately 150 and 230 nm, respectively. However, there was no significant difference in the particle size between the blank NPs and the Sal-loaded NPs (data not shown). This might be because the hydrophobic drug of Sal can be well encapsulated and equally distributed in the hydrophobic core of the NPs [38-40]. As shown in Figure 3, TEM depicted the shape of NPs prepared by these two methods with or without Sal. As we can see, the blank NR-NP and SE-NP also exhibited a spherical shape compared with the Sal-loaded NR-NP and SE-NP. We can distinguish the dark area in Sal-loaded NR-NP and SE-NP. This result helped us to deduce that Sal has been encapsulated into the NPs. The diameter observed by TEM was smaller than that detected on the Zetasizer Nano ZS Analyzer by DLS technique. There is a reason that the diameter of the NPs obtained by DLS reflected the hydrodynamic diameter of NPs swelling in water, while that observed by TEM was the diameter of dried NPs.

**Table 1:** The diameter and polydispersity of NPs prepared by different methods

**Nanoparticles**	**Diameter (nm)**	**Polydispersity**
Sal-loaded SE-NPs	235.8 ± 1.9	0.160 ± 0.028
Blank SE-NPs	224.1 ± 7.5	0.166 ± 0.005
Sal-loaded NR-NPs	151.1 ± 1.1	0.099 ± 0.041
Blank NR-NPs	148.1 ± 2.9	0.11 ± 0.015

Sal, salinomycin; SE-NPs, nanoparticles prepared by single emulsion method; NR-NPs, nanoparticles prepared by nanoprecipitation method.

### Drug loading content and encapsulation efficiency

The results of Sal encapsulation levels are shown in Table 2. SE-NPs exhibited higher entrapment efficiency than NR-NPs; this just corresponds to the results confirmed by several researchers [41,42]. We thought that this may be due to the larger size of SE-NP than NR-NP which could support a larger space for Sal encapsulation. In addition to this, the lower encapsulation efficiency of NR-NPs may partially be ascribed to the residue surfactant molecules on the particle surface, which were not washed away [43].

**Table 2:** The drug loading content and encapsulation efficiency of Sal NPs prepared by different methods

	**Drug loading content (%)**	**Encapsulation efficiency (%)**
Sal-loaded SE-NPs	89.70 ± 5.7	8.12 ± 5.7
Sal-loaded NR-NPs	81.51 ± 8.9	7.40 ± 8.9

Sal, salinomycin; SE-NPs, nanoparticles prepared by single emulsion method; NR-NPs, nanoparticles prepared by nanoprecipitation method.

### Stability evaluation

**Figure 4:**
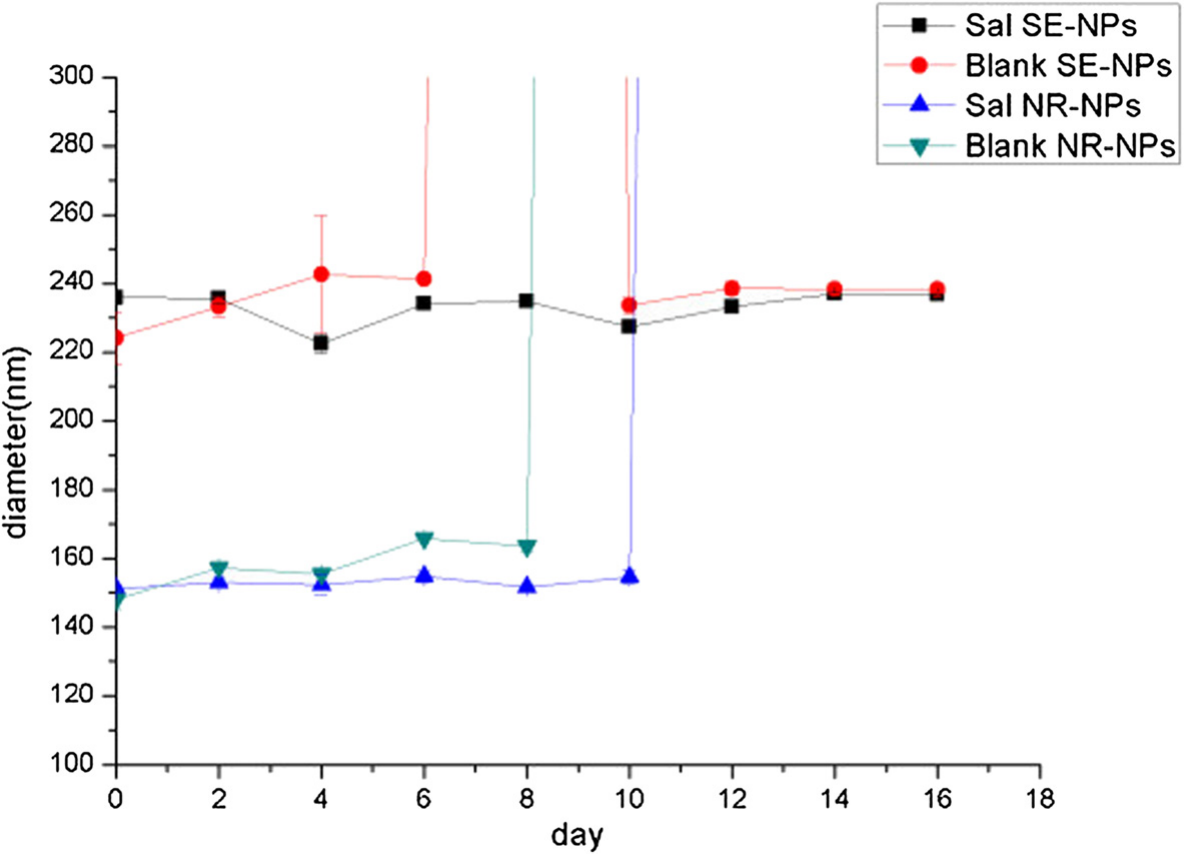
The stability of SE-NPs and NR-NPs.

Figure 4 shows the particle size change of NR-NPs and SE-NPs within 15 days [44,45]. During this period, the particle size of SE-NPs increased slightly. The particle size of NR-NPs with or without Sal existed with a large diameter in day 12; however, there were no obvious differences between the Sal-loaded NPs and blank NPs prepared by the nanoprecipitation method. Besides this, the particle size of NR-NPs decreased after 6 days in our observation. We consider that this may be because the NR-NPs were not stable enough; thus, most of the larger NPs aggregated, and the smaller NPs showed a decreased particle size. This result indicates that SE-NPs showed a better stability.

### In vitro release of Sal-loaded NPs

**Figure 5:**
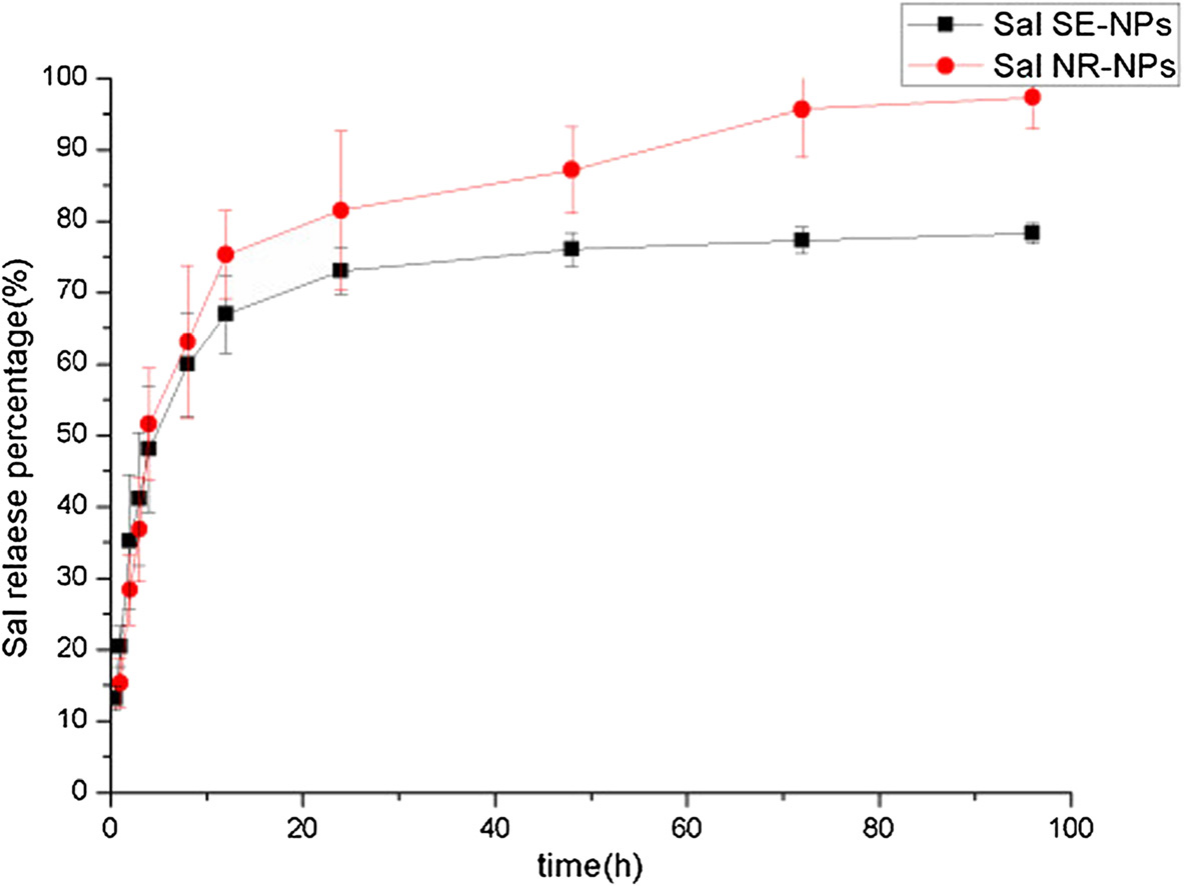
**The release curve of Sal from SE-NPs and NR-NPs.** Sal, salinomycin; NPs, nanoparticles; F68, poloxamer 188; SE-NPs, nanoparticles prepared by single emulsion method; NR-NPs, nanoparticles prepared by nanoprecipitation method.

To study the release behavior of Sal in vitro, SE-NPs and NR-NPs were incubated in a dialysis bag under shaking at 37°C, and the released Sal was quantified using pre-HPLC. Figure 5 shows the cumulative in vitro release of Sal from the two kinds of NPs. All NPs exhibited a fast release of Sal at the initial stage and a sustained release in the following time. NPs prepared by the nanoprecipitation method presented a more prominent burst release (approximately 56% at the first 4 h and 89% at the first 24 h). The release pattern of NPs prepared by the single emulsion method was more sustainable with a smaller initial burst rate (approximately 45% at the first 4 h and 70% at the first 24 h). This result may be due to the NR-NPs with the smaller particle size and surfactant molecules on the particle surface. In addition, Sal appeared to be released from Sal-loaded NPs in a biphasic way, that is, the release profile can be roughly divided into two phases: the first phase of release was complete within 12 h, considered as an initial burst, and the second phase showing sustained release for up to 96 h took place after this initial effect in which Sal was released.

### Primary toxicity observation of Sal-loaded SE-NPs

The number of living mice after injection with free Sal and Sal-loaded SE-NPs is shown in Table 3. According to the data that we collected, the mice died immediately after injection with free Sal (8 mg/kg). When half of the dose (4 mg/kg) was used, the mice did not die after injection. However, most of them died in the next day, and the number of viable mice was 2. Interestingly, when injected with Sal-loaded SE-NPs (8 mg/kg), none of the mice died in our observation period. Therefore, it was verified that encapsulating Sal into SE-NPs can reduce its side effects .The possible reasons for this are that (a) SE-NPs can target Sal delivery to tumor tissue which can increase the tumor's drug concentration and decrease drug concentration of normal tissues and (b) sustained release can keep the normal tissues out of lethal threshold.

**Table 3:** The number of live nude mice after injection with different drugs and dosages

	**Day 0**	**Day 1**	**Day 2**	**Day 10**
Free sal (8 mg/kg)	0	0	0	0
Free sal (4 mg/kg)	5	2	1	1
Sal-loaded SE-NPs (8 mg/kg)	4	4	4	4

Sal, salinomycin; SE-NPs, nanoparticles prepared by single emulsion method. Five mice in each group.

## Conclusions

In this paper, we used the PEG-Pep-PCL NPs, which have been proven effective for targeting tumor tissue specifically, to improve drug efficacy and reduce Sal-caused adverse effects. To determine the optimum method for Sal-loaded NP preparation, we made a comparison between NR-NPs prepared with different proportions of F68. Besides, we compared the physiochemical traits of NR-NPs and SE-NPs. Based on the results of our study, we confirmed that 1% is the best proportion of F68 for NR-NP preparation, due to the best stability of NR-NPs prepared with 1% of F68, and the SE-NPs showed superior stability, higher drug loading efficiency, and more sustainable but complete release, which means that the single emulsion method is better for Sal-loaded NP formulation. Preliminary toxicity observation indicated a striking higher survival rate of mice injected with Sal-loaded SE-NPs, which means that entrapping Sal into SE-NPs can greatly reduce its adverse effects. For consideration of the physical traits and in vivo animal study, we conclude here that SE-NPs prepared in this study have a high potential to be used for Sal delivery.

## Abbreviations

CSCs: cancer stem cells; Sal: salinomycin; EPR: enhanced permeation and retention effect; PVGLIG: gelatinase-cleavable peptide; NPs: nanoparticles; mPEG: polyethylene glycol; PCL: polycaprolactone; mPEG-NHS: methoxy-polyethylene glycol-NHS; DMF: dimethyl formamide; Et3N: triethylamine; DCM: dichloromethane; DMAP: 4-dimethylamiopryidine; EDC: 1-ethyl-3-[3-dimethylaminopropyl] carbodimide hydrochloride; NHS: *N*-hydroxysulfosuccinimide sodium salt; F68: poloxamer 188; PVA: polyvinyl alcohol; DLS: photon correlation spectroscopy; TEM: transmission electron microscope; DNP: 2,4-dinitrophenol; CDCl_3_: trichloromethane.

## Competing interests

The authors declare that they have no competing interests.

## Authors' contributions

QW carried out the preparation of nanoparticles and characterization of these nanoparticles. XJ, CX, and CY carried out the preparation of PEG-Pep-PCL copolymer. PW and LY carried out the HPLC. WR, QL, YY, and KX carried out the in vivo study. RL and LW conceived the study and participated in its design and coordination. BL helped to draft the manuscript. All authors read and approved the final manuscript.
